# The Lung, the Heart, the Novel Coronavirus, and the Renin-Angiotensin System; The Need for Clinical Trials

**DOI:** 10.3389/fmed.2020.00248

**Published:** 2020-05-22

**Authors:** Eugenie R. Lumbers, Sarah J. Delforce, Kirsty G. Pringle, Gary R. Smith

**Affiliations:** ^1^School of Biomedical Sciences and Pharmacy, University of Newcastle, Callaghan, NSW, Australia; ^2^Priority Research Centre for Reproductive Science, University of Newcastle, Callaghan, NSW, Australia; ^3^Pregnancy and Reproduction Program, Hunter Medical Research Institute, New Lambton Heights, NSW, Australia; ^4^VP System Practice, International Society for the System Sciences, Pontypool, United Kingdom

**Keywords:** renin-angiotensin system (RAS), ACE2, lung injury, cardiovascular disease, COVID-19, SARS- CoV-2

## Abstract

Angiotensin-converting enzyme 2 (ACE2) is the receptor for COVID-19 (SARs-CoV-2). ACE2 protects the lung and heart from acute respiratory distress syndrome (ARDS) and acute myocarditis and arrhythmias, because it breaks down Angiotensin II, which has inflammatory effects in the lung and heart as well as in the kidney. When SARS-CoV-2 binds to ACE2, it suppresses it, so this protective action of ACE2 is lost. Death from COVID-19 is due to ARDS and also heart failure and acute cardiac injury. Drugs that prevent the inflammatory actions of Angiotensin II (i.e., Angiotensin receptor blockers, ARBs) prevent acute lung injury caused by SARS-CoV. Clinical trials are underway to test the risks and benefits of ARBs and angiotensin-converting enzyme inhibitors (ACEIs) in COVID-19 patients requiring hospitalization. Other potential treatments are also discussed.

## Introduction

This article explains how the renin-angiotensin system (RAS) interacts with the severe acute respiratory syndrome coronavirus (SARS-CoV) and also with the novel coronavirus, SARS-CoV-2, which causes infection and subsequent acute lung and probably heart injury [COVID-19 (1–3)]. As well as identifying potential therapeutic strategies for treating acute lung injury and myocarditis in COVID-19 [see also (4–6)], this article provides a background to the management of patients with essential hypertension in accordance with recommendations made in the joint statement issued by the Heart Failure Society of America, the American College of Cardiology and the American Heart Association (7) that patients who are using drugs that block the RAS should continue to use them during this pandemic. It also describes why the use of angiotensin-converting enzyme inhibitors (ACEIs) and angiotensin receptor blockers (ARBs) is associated with less severe COVID-19 infections (8, 9).

**Figure 1:**
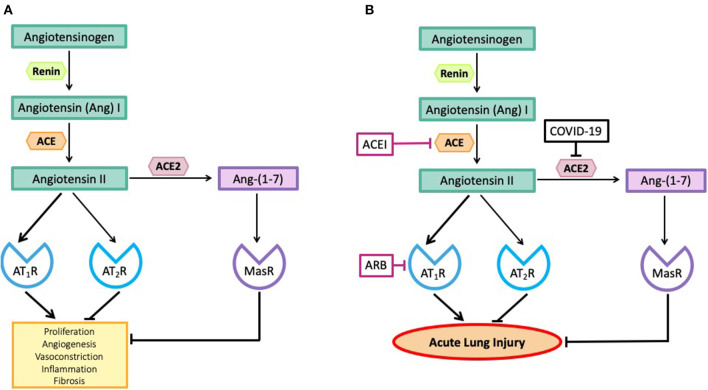
**(A)** The effects of angiotensin peptides on proliferation, angiogenesis, vasoconstriction, inflammation and fibrosis. Note the effects of Angiotensin II via the Angiotensin II type I receptor (AT_1_R) are blocked by its interaction with the Angiotensin II type II receptor (AT_2_R) and by its metabolism to Ang–(1-7) acting via the Mas receptor (MasR). **(B)** SARS-CoV-2, by inhibiting ACE2, blocks the metabolism of Angiotensin II to Ang–(1-7) so blocking the anti-inflammatory pathway and causing high levels of Angiotensin II. Angiotensin converting enzyme inhibitors (ACEIs) prevent the formation of Angiotensin II and angiotensin receptor blocking drugs (ARBs) prevent Angiotensin II from causing inflammation and fibrosis.

There are two major arms of the RAS, one arm, the Angiotensin II (Ang II)-Ang II type 1 receptor (AT_1_R) pathway is pro-inflammatory and can cause acute lung injury (10, 11). The other arm, the angiotensin-converting enzyme 2 (ACE2)-Ang–(1-7)-Mas receptor (MasR) pathway is anti-inflammatory because ACE2 metabolizes Ang II, thus reducing its levels and converting it to the anti-inflammatory peptide, Ang–(1-7) [Figure 1A, (2, 12)].

ACE2 is the receptor for coronaviruses, including SARS-CoV-2 (4). As a result of SARS-CoV-2 binding to ACE2, the enzyme is no longer functional (13). Thus, the pro-inflammatory Ang II-AT_1_R is no longer blocked by the ACE2-Ang–(1-7)-MasR pathway and it is this imbalance that causes acute lung injury (13). A multi-centered double blind clinical trial has recently been established to test the efficacy of treating patients suffering from COVID-19 with an ARB (https://clinicaltrials.gov/ct2/show/NCT04312009). A second is investigating the outcomes of treatment of COVID-19 patients with ACEIs or ARBs (https://clinicaltrials.gov/ct2/show/NCT04331574).

Coronaviruses are a group of viruses that have in recent years caused epidemics of acute respiratory syndromes. The first major epidemic was SARS (Severe Acute Respiratory Syndrome)-CoV in 2003 which was responsible for 8,000 deaths; the second was MERS (Middle East Respiratory Syndrome), which occurred in 2012. The most recent is SARS-CoV-2 which causes COVID-19. It was first recognized in China in December 2019 and is now sweeping the world. SARS-CoV-2 is already responsible for more cases of infection and also more deaths than the two previous coronavirus epidemics. There are also a number of other coronaviruses that cause upper respiratory tract infections, in particular HCoV-NL63.

SARS-CoV and SARS-CoV-2 both enter the cell by binding to ACE2 (13, 14), as does HCoV-NL63 (15). HCoV-NL63, like SARS-CoV-2, can cause mild respiratory infections, but most commonly affects the young (16). It has, however, been associated with bronchiolitis and croup but does not cause the acute respiratory distress syndrome (ARDS) characteristic of SARS-CoV-2 infection. The differences in the severity of illness caused by these two viruses could be related to a differing affinity of the viruses to ACE2 (17) or the fact that entry of HCoV-NL63 into cells requires intracellular acidification while SARS-CoV entry can occur independent of intracellular acidification (18). MERS-CoV binds to a different receptor, Dipeptidyldipeptidase4 (DPP4) (19), and another human coronavirus, HCoV-229E, uses aminopeptidase N (APN) as its receptor (20). These receptors are membrane-bound proteases, and all can affect the production or metabolism of angiotensin peptides (21, 22).

Zhang et al. in a clinical study of 140 patients found that hypertension and diabetes were the two most common comorbidities in patients with COVID-19 (23). The increase in the prevalence of these two comorbidities in patients with severe disease was not significant when compared with those with infections that were not severe (23). In a study by Guan et al. 261 patients (23.7%) had a co-existing disorder and 21.5% of them reached the composite end-point (admission to ICU, use of mechanical ventilation or death) (24). Of the 15% that had hypertension, and 7.4% that had diabetes, 13.1 and 6.1% (respectively), of these patients reached the composite end-point. It should be noted that hypertension and diabetes are very common comorbidities, no other co-existing disorders were as prevalent in this cohort.

There has been considerable debate about the use of drugs that block the RAS in the treatment of hypertensive patients who have COVID-19 (25). This debate has not adequately considered the roles of local pulmonary and circulating RASs in the pathogenesis of COVID-19. Put simply, ACE2, the receptor for entry of SARS-CoV and SARS-CoV-2, also activates a RAS pathway that prevents acute lung injury (1, 2). These ‘Ying' and ‘Yang' actions of ACE2 have caused an apparent dilemma concerning the use of RAS blocking drugs in the treatment of hypertension and diabetes. This is because while they upregulate the SARS-CoV-2 receptor (ACE2) (a means for viral entry into cells), they also protect tissues from the pro-inflammatory actions of Ang II and could be an effective therapeutic strategy to manage COVID-19 induced lung injury [see also (4, 5)]. This seems to be the case in the light of recent clinical studies (8, 9).

## The Renin-Angiotensin System (RAS)

Figure 1A shows the major arms of the RAS that are involved in the pathogenesis of coronavirus-induced acute lung injury. We have avoided including a number of other RAS pathways involved in the metabolism of Ang II for simplicity. A more thorough description of these pathways can be obtained elsewhere (12). As seen in Figure 1B, there are two major pathways involved in the pathogenesis of coronavirus-induced infections. Both pathways involve the formation of Ang II from Ang I, a peptide produced by the action of renin on angiotensinogen. The octapeptide, Ang II, is formed by the action of angiotensin converting enzyme (ACE) on Ang I. Ang II can bind to two receptors, the AT_1_R and the AT_2_R, or it can be broken down by number of proteases to smaller peptides that have a variety of biological actions.

The most important pathway for Ang II break down is via the removal of one amino acid from its C-terminus by a zinc-dependent carboxy peptidase, ACE2, to generate the peptide, Ang–(1-7), which acts on another receptor known as the MasR. There are other pathways that can also generate Ang–(1-7) (12). These two RAS pathways (Ang II-AT_1_R) and ACE2-Ang–(1-7)-MasR have opposing actions. In addition, Ang II can act via the AT_2_R to produce effects similar to those generated by the ACE2-Ang (1-7)-MasR pathway (12).

The Ang II-AT_1_R axis is well-known because it is responsible for hypertension; it raises blood pressure both through actions in the brain on the sympathetic nervous system, and in peripheral blood vessels causing vasoconstriction. This axis also controls sodium reabsorption partly through its own actions in the kidney but also because it stimulates the release of the sodium retaining hormone, aldosterone, from the adrenal gland.

There are other actions of this pathway that are pro-inflammatory, and which stimulate fibrosis. The ACE2-Ang–(1-7)-MasR pathway on the other hand lowers blood pressure, possibly through production of nitric oxide. Furthermore, the ACE2-Ang–(1-7)-MasR pathway is anti-inflammatory. Thus, ACE2 not only breaks down Ang II but also produces a vasodilator anti-inflammatory molecule, Ang–(1-7) (26).

## Circulating and Tissue RASS

The circulating RAS is an endocrine system capable of reaching the brain, the heart and the lungs but there are also local tissue RASs, in many organs, such as the heart, the kidney, the female reproductive tract, and the brain. There is also a local lung RAS (27) that has been implicated in the etiology of pulmonary fibrosis (11, 28).

Through a combination of circulating and tissue systems, the RAS can have powerful effects in the lungs. Renin is released from the kidneys into venous blood and renal lymphatics. It produces Ang I from angiotensinogen, which is produced by the liver. Ang I in the venous blood returning to the heart and lungs is converted in the lung by ACE to Ang II. Thus, the lung is exposed to high levels of Ang II.

## ACE2

The gene for ACE2 is located on Xp22 and contains 18 exons, many of which are similar to the ACE gene. It is a Zn carboxypeptidase with only one catalytic site, and it has 40% homology with ACE. It is an ectoenzyme with its N-terminus and catalytic site facing the extracellular space so it can metabolize circulating peptides (29). ACE inhibitors (ACEIs) do not affect its activity.

The spike (S) protein on the surface of the SARS-CoV-2 (COVID-19) mediates receptor recognition and membrane fusion. The spike protein is trimeric and, on fusion, separates into S1 and S2 subunits. S1 contains the receptor binding domain that directly binds to the catalytic site of ACE2. When this occurs a cleavage site on the S2 protein develops which is acted on by host proteases resulting in membrane fusion (14). The S protein of SARS-CoV-2 binds to the catalytic site of ACE2 more efficiently than does the SARS-CoV (30); unfortunately antibodies that recognize the SARS-CoV receptor binding domain (RBD) do not recognize the SARS-CoV-2 RBD (30). However, sera from convalescent SARS patients cross neutralized the SARS-spike protein (S)-driven entry into cells (31) and polyclonal murine antibodies directed against the SARS-CoV spike protein (S) potently inhibit SARS-CoV-2 S cell entry (32).

There is a widespread distribution of ACE2 throughout the body (33), including the lungs (11), heart (34, 35), and kidney (36).

Coronaviruses gain access to the body via the respiratory tract and it has recently been shown that nasal goblet cells, type II pneumocytes and ileal enterocytes all possess the necessary combination of ACE2 and TMPRSS2 for viral entry to be successful (31, 37). HCoV-NL63 binds to human airway epithelial cells *in vitro* (38). In the lung, ACE2 is widely distributed throughout the bronchial and pulmonary epithelium and the pulmonary capillaries (39). ACE2 protects the lung from the pro-inflammatory and pro-fibrotic actions of circulating Ang II by metabolizing Ang II to Ang–(1-7), which, acting via the MasR, inhibits Ang II-AT_1_R pro-inflammatory pathways [Figure 1A, (4)].

ACE2 levels vary with age, being highest in young animals and lowest in older animals, levels in older males and females being 78 and 67% lower, respectively (40). This suggests that young people are more likely to get the SARS coronavirus infection than older people, as appears to be the case. On the other hand, decreased production of ACE2 in the elderly could be one reason why coronaviruses cause more serious complications in older persons. It has to be said however, that there has not been a systematic study of the relationships between age and ACE2 expression in human tissues and, as explained below, there may be species specific differences. In the lungs (1, 2), the heart (35, 41), and the kidney, ACE2 (36) protects against the pro-inflammatory actions of Ang II acting via the AT_1_R.

## Drugs That Block The RAS

Figure 1B shows two sites of action of drugs that block the activity of the RAS mediated by the interaction of Ang II with the AT_1_R. One of the two classes of drugs commonly used in clinical practice, blocks the activity of the RAS by blocking the formation of Ang II; it inhibits the activity of ACE. ACE is a Zn containing carboxypeptidase that removes a dipeptide from the C-terminal end of the decapeptide, Ang I, thus generating the active peptide responsible for most actions of the RAS, Ang II. Drugs that block this enzyme are known as ACE inhibitors (ACEIs). They have no direct effect on the activity of ACE2, except by limiting the amount of Ang II that is produced.

The second group of drugs are known as angiotensin receptor blockers (ARBs); they block the interaction of Ang II with the AT_1_R. ARBs do not reduce Ang II levels.

The use of these drugs could result in two significant consequences:

1. ARBs cause a rise in Ang II levels. This increase in Ang II results from blocking Ang II's effects on blood pressure and sodium and water balance and indirectly causing positive feedback on renin release. High levels of Ang II will result in increased conversion of Ang II to Ang–(1-7) by ACE2 and increased interaction of Ang II with the AT_2_R. This means that not only are the pro-inflammatory effects of Ang II-AT_1_R prevented but the anti-inflammatory effects mediated by the ACE2-Ang–(1-7)-MasR axis are enhanced and Ang II-AT_2_R's anti-inflammatory effects are also sustained. As well, it has been shown in the heart that ARBs cause upregulation of ACE2 because they prevent Ang II-AT_1_R mediated reductions in ACE2 activity (42). It is not known however, if this up-regulation of ACE2 occurs in the lungs.
2. ACEIs block the formation of Ang II, so there is no associated enhancement of any anti-inflammatory effects mediated by the ACE2-Ang–(1-7)-MasR axis and the AT_2_R nor would there be up-regulation of ACE2. There is only withdrawal of Ang II-AT_1_R's pro-inflammatory actions.

## Lung ACE2 and Coronaviruses

Lung ACE2 is the receptor for both SARS-CoV and SARS-CoV-2 (13, 31). The spike (S) protein binds to ACE2 and enters the cell where it is modified by a serine protease (TMPRSS2). This protease is essential for cell entry of the virus (31). The binding of coronaviruses to ACE2 and the modification of its spike protein by TMPRSS2 are essential for infection.

Binding of the SARS-CoV spike protein to ACE2 results in reduced ACE2 protein levels (13). Kuba et al. (13) showed that *ACE2*-/- knockout mice did not get infected with SARS-CoV and did not get acute lung inflammation (13). Thus, it would appear that low levels of pulmonary ACE2 protect against coronavirus infection. There is however a sinister side effect to the loss of pulmonary ACE2 because it plays a critical role in preventing acute lung injury.

Xie et al. suggest that there is a greater prevalence of SARS-CoV in young people because they have higher ACE2 levels (40). Yet there is a paradox, because if young people, like other mammals, have high levels of pulmonary ACE2, they should be more susceptible to symptomatic infection with coronaviruses. The prevalence of infection is however based on the appearance of symptoms and in the young, the disease is usually so mild that infection rates appear to be low. In fact, the milder nature of the disease in the young compared to the old could be a consequence of the age-dependent nature of ACE2 expression in the lung (see above). On the other hand it is possible that the animal data on ACE2 expression and age is misleading as some human studies have shown that the older the patient the higher the level of circulating ACE2 (43, 44). As stated above, there is no systematic study exploring the effects of age on ACE2 expression by human tissues. The differences between animal-based studies and human data may well be related to the short life span of animals used and the fact that they are housed in environments that protect them from infections and pollution etc.

It has also been claimed that ARBs stimulate the expression of ACE2 in the lung and that this accounts for a higher morbidity in hypertensive patients suffering from COVID-19 (25). As stated above, there is no evidence that ARBs have this effect on pulmonary ACE2, but ARBs upregulate cardiac ACE2. Ang II levels are likely to be elevated in patients treated with an ARB. As Ang II is the major substrate from which ACE2 produces Ang–(1-7) (1), it might be expected that high levels of Ang II would upregulate ACE2. However, Ang II and Ang–(1-7) have counter regulatory actions on ACE2 expression via MAP kinase/phosphatase pathways (45). Furthermore, Ang II downregulates ACE2 activity in cardiac myocytes and fibroblasts (42). Therefore, the overall effects of ARBs on pulmonary ACE2 could be modified by counteracting the effects of Ang–(1-7).

Hypertension and diabetes are the most common comorbidities found in patients suffering from COVID-19. In one study, there was no significant difference in the existence of either comorbidity between less severe and more severe cases (23). However, in a study comparing 113 patients who died with COVID-19 infections, hypertension and cardiovascular disease were more common than in patients who recovered (161). Furthermore, acute cardiac injury and heart failure, like acute respiratory distress syndrome and respiratory failure, contributed to the critical nature of the illness (46).

## The Role of ACE2 In Protecting The Lung and Heart From ANG II Induced Inflammation

ACE2 protects the lung from acute lung injury because it reduces levels of Ang II by converting it to Ang–(1-7). In 2005, Imai et al. (1) showed that ACE2 played a critical role in the prevention of lung injury. Briefly, they induced acute lung injury in mice by sepsis, lipopolysaccharide (LPS) endotoxin or by acid aspiration. These treatments all caused severe lung inflammation in *ACE2*-/- knockout mice, which was mitigated by intraperitoneal injections of recombinant human ACE2 (1). In a second publication in 2005, Kuba et al. (13) not only showed that ACE2 was the definitive receptor for SARS-CoV, but that loss of ACE2 from the lung caused by the binding of viral spike protein was responsible for the acute lung injury caused by coronavirus infections. They also showed, as would be expected from the known actions of ACE2, that this viral infection was associated with raised Ang II levels, which caused acute lung injury via the Ang II-AT_1_R pathway. Treatment with the ARB, losartan, prevented SARS-CoV-induced lung injury (13).

We suggest that pulmonary ACE2 plays a critical role in protecting the lung from Ang II-AT_1_R induced inflammation because not only is there a local pulmonary RAS but the lung is also the major site for conversion of Ang I (which is inactive) to Ang II (47). Therefore, loss of ACE2, by binding of SARS-CoV-2, not only exposes the pulmonary epithelium to locally formed Ang II but also to Ang II formed in the lung from circulating Ang I.

Recently, a SARS-CoV-2 infected patient presented with acute heart failure a week after experiencing “flu-like symptoms” and was diagnosed with acute myocarditis without any interstitial pneumonitis (48). It is known that SARS binds to myocardial ACE2 and downregulates myocardial ACE2 protein (3). The protective effects of ACE2 in the heart are well-described (41, 49) and it is the major metabolic pathway for breakdown of Ang II in the heart (50). ACE2 protects the heart from Ang II-AT_1_R signaling induced injury (34).

## Therapeutic Modalities For The Treatment of Coronavirus-Induced Lung Injury

To summarize; the data described above demonstrate that there could be a problem in treating coronavirus infections. While it is most advisable to prevent viral infection and reduce the viral load, prevention of coronavirus-associated lung and cardiac injury saves lives. The dual role of ACE2 as a receptor for the virus in the lungs and heart but a “protector” of the lungs and heart from coronavirus-induced injury has led to debate concerning the use of anti-hypertensive drugs that inhibit the RAS because they upregulate ACE2 in the lung, increase receptor availability and therefore may increase viral load.

There is no doubt, however, that Ang II-acting via the AT_1_R causes acute lung injury and probably cardiac injury when the ACE2-Ang–(1-7)-Mas receptor pathway is blocked by downregulation of ACE2. This pathway also protects from other forms of acute lung injury as well as SARS-CoV induced injury. ARBs would prevent downregulation of this pathway once infection has occurred and ameliorate any unopposed pro-inflammatory effects of Ang II mediated via its AT_1_R. ACEIs on the other hand may not upregulate ACE2 but would prevent Ang II-induced inhibition of ACE2 activity.

## Preventing Coronavirus Infections In The Lung

Since there is an approved serine protease inhibitor that could be used to block TMPRSS2, it is possible that this could be used to treat COVID-19. Another potential alternative could be to saturate the airways with recombinant ACE2 or soluble ACE2 so that viral particles are “mopped up” leaving bronchial pulmonary ACE2 intact. Treatment with recombinant human ACE2 could have the added beneficial effect of protecting the lung against lung injury.

## Treating Coronavirus-Induced Lung Injury

The major problem facing treatment of coronavirus-induced lung and probably cardiac injury is that there is reduced ACE2 caused by the virus binding to ACE2. This means that Ang II is no longer metabolized by ACE2, and the anti-inflammatory Ang–(1-7) pathway is lost. Thus, lung and cardiac Ang II-AT_1_R pro-inflammatory pathways are activated and unopposed by the protective arm of the RAS. As suggested by Kuba et al. (13), ARBs are an appropriate adjunct therapy for treating coronavirus-induced lung injury. Recent analysis of clinical data from patients with COVID-19 support the use of RAS blocking drugs in the treatment of this infection (8, 9). Other alternatives, as suggested by Imai et al., include injection of recombinant ACE2. Haschke et al. (51) have shown the rhACE2 is well-tolerated by healthy human subjects.

The publication of the first clinical trials to test the efficacy of ARBs and ACEIs in the treatment of acute lung injury induced by SARS-CoV-2 is welcome news because these drugs are widely used clinically. If the results of these trials support the clinical data already obtained and improve the outcome of coronavirus infections, they would be of immeasurable benefit in the clinical management of this pandemic.

## Author Contributions

EL wrote the first draft of the manuscript. All authors contributed to manuscript revision, read and approved the submitted version.

## Conflict of Interest

The authors declare that the research was conducted in the absence of any commercial or financial relationships that could be construed as a potential conflict of interest.
